# Epsilon-poly-L-lysine: Recent Advances in Biomanufacturing and Applications

**DOI:** 10.3389/fbioe.2021.748976

**Published:** 2021-09-28

**Authors:** Liang Wang, Chongyang Zhang, Jianhua Zhang, Zhiming Rao, Xueming Xu, Zhonggui Mao, Xusheng Chen

**Affiliations:** ^1^ The Key Laboratory of Industrial Biotechnology, Ministry of Education, School of Biotechnology, Jiangnan University, Wuxi, China; ^2^ School of Food Science and Technology, Jiangnan University, Wuxi, China; ^3^ State Key Laboratory of Food Science and Technology, Jiangnan University, Wuxi, China

**Keywords:** epsilon-poly-l-lysine, strain improvement, process engineering, downstream process, antimicrobial property

## Abstract

ε-poly-L-lysine (ε-PL) is a naturally occurring poly(amino acid) of varying polymerization degree, which possesses excellent antimicrobial activity and has been widely used in food and pharmaceutical industries. To provide new perspectives from recent advances, this review compares several conventional and advanced strategies for the discovery of wild strains and development of high-producing strains, including isolation and culture-based traditional methods as well as genome mining and directed evolution. We also summarize process engineering approaches for improving production, including optimization of environmental conditions and utilization of industrial waste. Then, efficient downstream purification methods are described, including their drawbacks, followed by the brief introductions of proposed antimicrobial mechanisms of ε-PL and its recent applications. Finally, we discuss persistent challenges and future perspectives for the commercialization of ε-PL.

## Introduction

ε-poly-L-lysine (ε-PL) is a homopoly(amino acid) consisting of 25–35 L-lysine residues with amide linkages formed between the ε-amino and α-carboxy groups. Since it has many valuable characteristics such as bacteriostatic, soluble, biodegradable, edible, and non-toxic to humans and environment, ε-PL is widely used in food and medicine industries as food preservatives, dietary agents, and gene/drug/vaccine carriers (Shih et al., 2006; Yuan et al., 2018). In 1989, ε-PL was first approved as a natural food preservative by the Ministry of Health, Labour and Welfare in Japan. Then it has been used as a preservative in many countries such as South Korea, United States and China (Xu et al., 2016).

ε-PL can be synthesized chemically *via* many methods, such as solid-phase synthesis and ring-opening polymerization (Tao et al., 2015; Tao, 2016). However, these methods often contain complicated steps (e.g., protection and deprotection reactions) with low ε-PL yield, which may generate a lot of by-products and raise environmental issues. Compared with chemosynthesis, microbial production is a more attractive approach for ε-PL industrialization because it is more economic, practical, efficient and environmentally friendly. Currently, a total of only three companies around the world have been able to manufacture ε-PL at commercial scale, which are Chisso Co. (Tokyo, Japan), Okuno chemical industries Co. (Osaka, Japan) and SunBio Co. (Shizuoka, Japan). Among them, Chisso Co. was the most prominent ε-PL manufacturers, which established a production line with an annual output of 1,000 tons, using a mutant strain of *Streptomyces albulus* (Hirohara et al., 2007). These three companies have provided the majority of ε-PL in the world at a relatively high price (180 USD/kg). As a novel value-added product, the growing demand for ε-PL has facilitated related researches in biomanufacturing and industrial applications.

**FIGURE 1:**
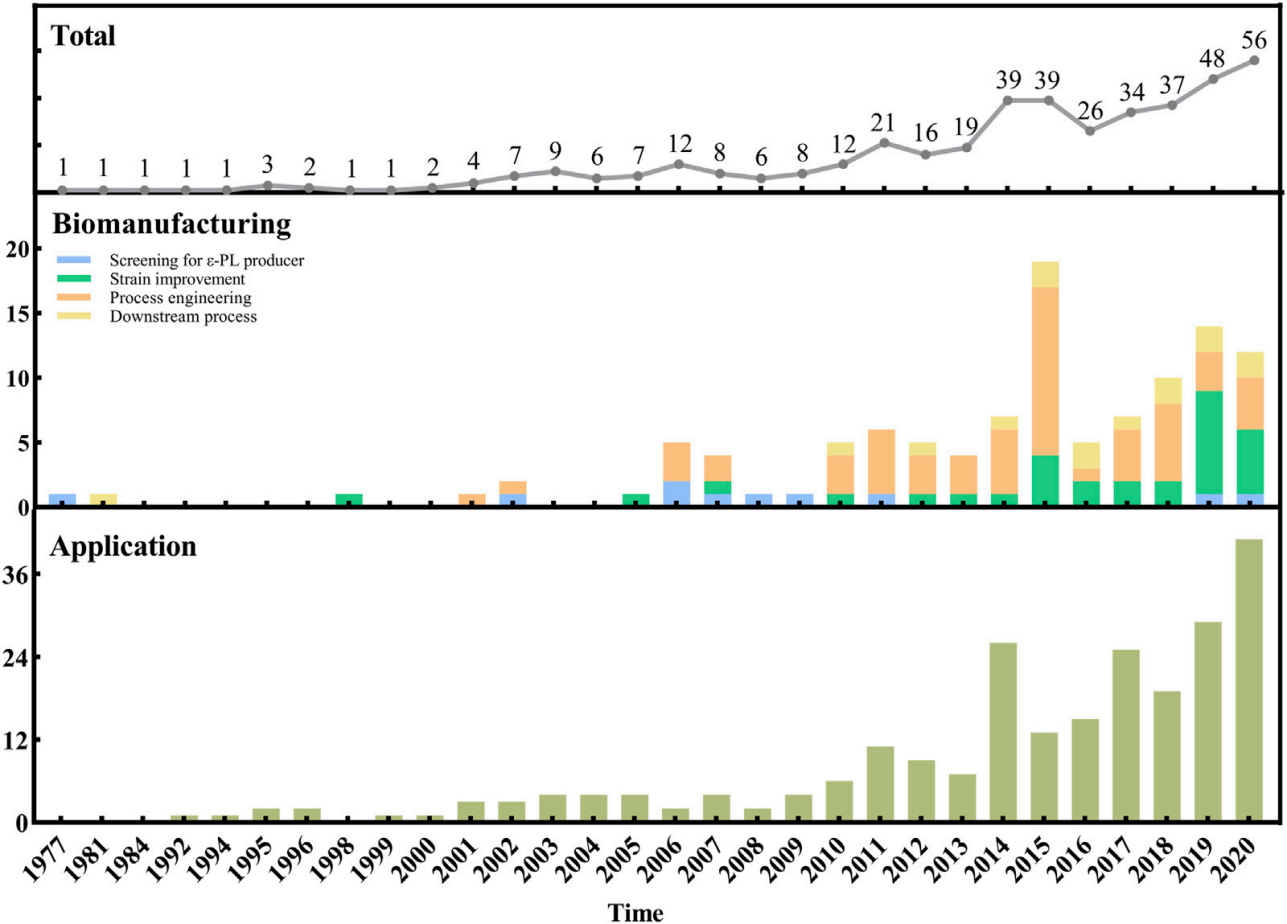
Evolution over the years of the number of references concerning the biomanufacturing and applications of ε-PL.

Figure 1 provides a holistic overview of ε-PL research beginning with the year when ε-PL was discovered. From 1977 through 2009, research efforts were relatively low and focused mostly on methods for isolation of ε-PL-producing strains. After that, research has increasingly focused on strain improvement and process engineering for enhancing the ε-PL production. It can also be noted that studies concerning downstream process have been few but constant during the last 7 yr. In addition, the literature on ε-PL applications also significantly increased since 2014, which may be related to the increasing number of breakthroughs in ε-PL biomanufacturing (e.g., the improved ε-PL production and the reduced cost).

In recent years, substantial progress has been made in the biomanufacturing of ε-PL, including: *1*) recent technologies (e.g., gene mining) developed for screening novel ε-PL producers, other than filamentous microorganisms; *2*) some novel methods (e.g., gene swap strategy) for controlling the distribution of ε-PL molecular weight; *3*) some recent strategies (e.g., ribosome engineering) and genetic engineering methods for directed evolution of robust ε-PL-producing strains; *4*) economical and high efficiency techniques for ε-PL production, such as low-cost feedstocks for fermentation, solid-state fermentation, *in situ* product removal, and cell immobilization, and *5*) different downstream extraction processes optimized for higher purity and recovery at relatively lower cost. Moreover, since many applications of ε-PL in food and pharmaceutical industries are dependent on its antimicrobial capacity, the understanding of the action mode of ε-PL on different microorganism is helpful in guiding its applications. This review first summarized and discussed the recent advances in ε-PL biomanufacturing, focusing on the strain improvement and process engineering. Subsequently, the latest studies concerning applications of ε-PL were briefly introduced, including its antimicrobial mechanisms. Finally, we conclude with discussion of the existing challenges and future perspectives for this burgeoning field. We hope this review can help to promote the development of ε-PL-producing strains and the integrated, large-scale production (and downstream) processes of ε-PL for various industrial applications.

## Biosynthesis of ε-PL by Various Microorganisms

### Typical ε-PL-producing Strains

In 1977, ε-PL was accidentally discovered as an extracellular secretion produced by *S. albulus* NBRC14147 (Shima and Sakai, 1977)*.* Following this discovery, no ε-PL-producing microbe was isolated until a dye-based method was developed in 2002 (Nishikawa and Ogawa, 2002). A total of 10 ε-PL producers were isolated from 300 soil samples *via* this easy and efficient method, and were identified as members of the genera *Streptomyces*, *Epichloë, Streptoverticillum*, *Kitasatospora*.

Currently, increasing numbers of ε-PL-producing strains have been screened out as the development of novel screening methods. Efforts in ε-PL biomanufacturing research have mainly focused on *Streptomyces*, especially on *S. albulus*, which gives the highest reported ε-PL production (70 g/L) in liquid fermentation after strain evolution and fermentation process optimization (Wang et al., 2019). Some other actinomycetes, such as *Kitasatospora*, *Streptomyces ahygroscopicus*, *Streptomyces griseofuscus* and *Streptomyces diastatochromogenes* have also been investigated in some studies, with various degrees of success (Ouyang et al., 2006; Chen J. et al., 2019; Shu et al., 2019; Wang et al., 2021). Additionally, in 2012, a marine *Bacillus subtilis* SDNS was first discovered to produce a small amount of ε-PL (76.3 mg/L) under the optimized culture conditions (El-Sersy et al., 2012). Subsequently, some other bacteria such as *Bacillus thuringiensis*, *Bacillus cereus*, *Bacillus licheniformis*, *Lactobacillus delbrueckiistrain, Lactococcus lactisstrain* and *Corynebacterium variabile* were also identified to have ε-PL synthetases in their genomes, indicating that bacteria also have the potential to be industrial ε-PL producers (Shukla and Mishra, 2013; Chheda and Vernekar, 2014; Bhattacharya et al., 2017; Samadlouie et al., 2020; Jiang et al., 2021). Recent study also showed that ergot fungi (*Epichloë festucae*) can produce higher ε-PL production (69.9 mg/L) *via* genetic engineering (Purev et al., 2020). The discovery of ε-PL synthetases in these microbes suggests that ε-PL can be found naturally not only from soil samples under decayed plant matter, but also in marine water, entomo, plants, cheese, or even on human skin, and play a role in their ecologies (Chheda and Vernekar, 2014; Purev et al., 2020; Jiang et al., 2021). With the constant innovation of methods for isolation, it is believed that a greater variety of ε-PL producers will be discovered for industrial production, with a wider distribution.

### Screening for ε-PL Microbes

Screening for ε-PL microbes has recently attracted considerable research attention. However, the proportion of these strains in nature is relatively small, and there are no distinct characteristics for direct identification of ε-PL-producing microbes. Historically, the conventional method for screening ε-PL producers is to analyze extracellular secretions, although this technique is unsuitable for large culture collections.

An efficient, commonly used method for screening isolates is by detecting the interaction between basic/acidic dyes and polycationic ε-PL secreted in culture broth. For example, Nishikawa and Ogawa (2012) incubated strains in a agar culture media containing either basic (methylene blue) or acidic dyes (Poly R-478), then picked potential ε-PL-producing isolates that formed haloes in the medium. This method has been used to obtain large numbers of new candidate isolates, especially members of two group of soil microflora, Streptomycete and ergot fungi (Nishikawa and Ogawa, 2002). Although such a method was determined to be accurate and sensitive, the toxicity of methylene blue can affect the detection rate of ε-PL-producing microbes. To address this issue, Li et al. (2011) proposed a “sandwich layer” method in which colonies from soil samples are first cultured on medium A, then the whole agar of medium A is lifted from the plate and transferred to the surface of medium B (containing the methylene blue indicator), thus forming a medium A-colonies- medium B “sandwich.” After incubation for 2 h at 40°C, strains forming haloes are then picked and purified. This method was successfully used to identify *S. griseofuscus,* which can yield 7.5 g/L ε-PL in fed-batch fermentation (Li et al., 2011).

“Two-stage culture” is another commonly used method for strain screening. Shima et al. (1983) proposed this method based on the discrepancy between the optimum pH of cell growth and ε-PL accumulation in *S. albulus* No. 346. Subsequently, Hirohara et al. (2006) developed the two-stage culture method in which each colony was first cultured in growth medium (pH 6.8) for 20–48 h (cell growth culture stage), then mycelium was collected, washed, resuspended, and incubated in production medium (ε-PL production culture stage) (Hirohara et al., 2006). The two-stage culture method is used extensively and many ε-PL producers have been successfully isolated and tested, such as *Streptomyces* sp. USE-11 and *Kitasatospora* sp. PL6-3 (Ouyang et al., 2006; Hirohara et al., 2007).

The “half-leaf” method is a recently reported protocol for screening ε-PL-producing actinomycete strains (Chen X. et al., 2019). The principle underlying this method is that ε-PL demonstrates an obvious protective and curative activity against Tobacco mosaic virus (TMV). Thus, the left side of a *Nicotiana glutinosa* leaf is treated with TMV and the secretion of *Streptomyces* strains, while the right side of the same leaf is treated only with TMV. By comparison of the numbers of local lesions on left and right side of *N. glutinosa* leaves, *S. ahygroscopicus* STZ was identified as an ε-PL producer.

Genome mining technique have also come into wide use for high-throughput screening for strains that carry genes encoding putative Pls. Homologs of Pls in different species can show low sequence similarity, and hence, some ε-PL coding sequences have been overlooked during the gene annotation. *S. albulus* Pls is the most thoroughly studied Pls, and its homologs in Streptomycetes are well conserved. Geng et al. (2014) revealed that *pls* gene sequences from *S. albulus* NK660 showed 99 and 84% similarity with that from two other Streptomycete strains (*S. albulus* NBRC14147 and *S. roseoverticillatus* MN-10). Recently, a gene encoding a Pls-like protein was discovered in the genome of *C. variabile* by NCBI BLAST search, and although the similarity between this protein and Pls from *S. albulus* is only 51%, they share highly similar domain architecture (Jiang et al., 2021). This method also facilitated identification of several Pls homologs in the genomes of Coryneform actinobacteria (i.e., *Brevibacterium*, *Arthrobacter,* etc.) and Ascomycota fungi (Purev et al., 2020; Jiang et al., 2021).

In summary, there are several methods for successful screening of ε-PL-producing strains, including dye-based indicators, two-stage culture, and genome mining. Dye-based methods are straight forward but laborious, while “two-stage culture” and genome mining are more user-friendly, but require several time-consuming steps of mycelium cultivation and product detection or DNA sequencing and BLAST analysis, respectively. A more direct and simpler method, such as detection by HPLC, may also serve as a viable option for identification of ε-PL-producing strains.

### Key Enzymes in ε-PL Biosynthesis Pathway

**FIGURE 2:**
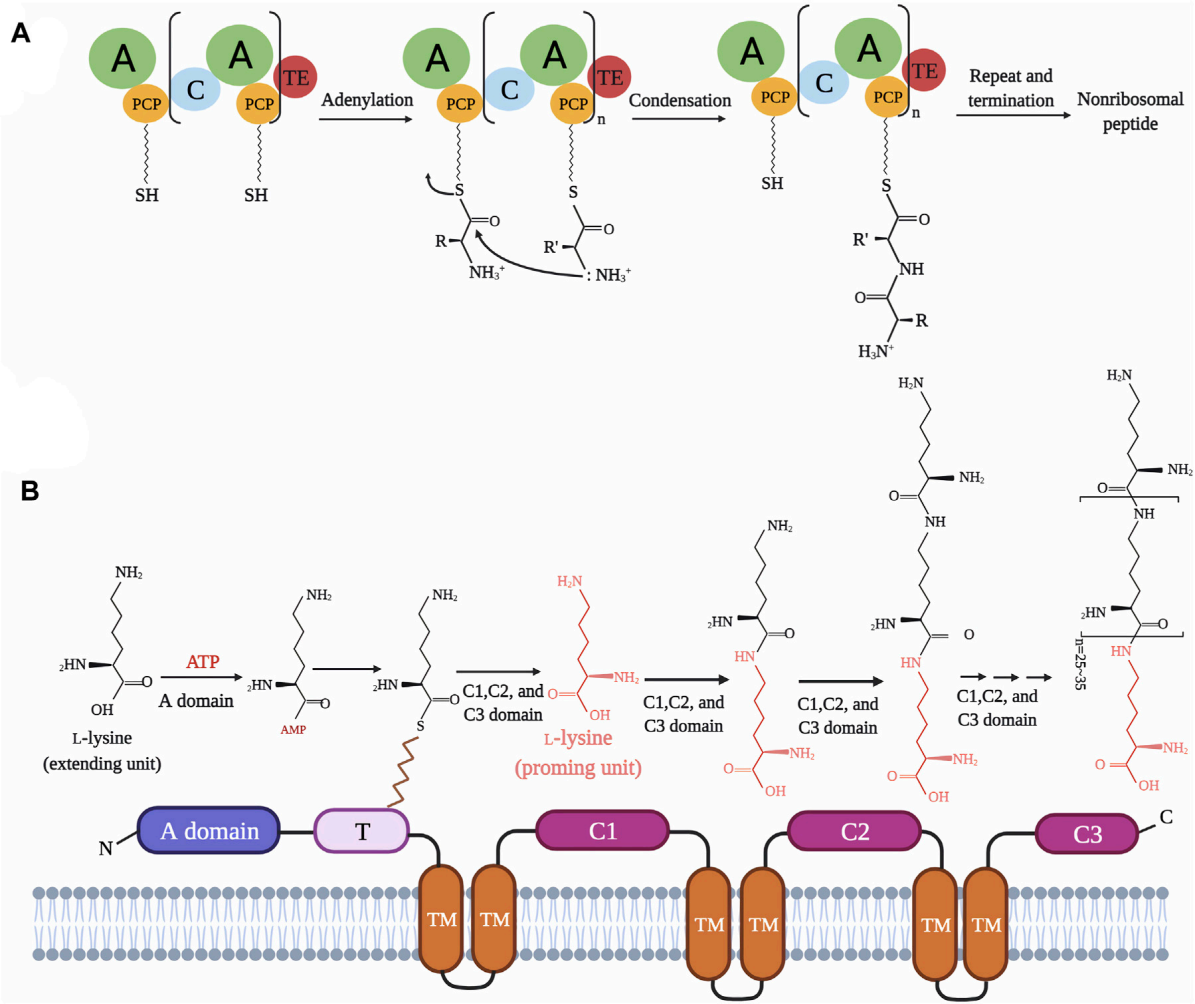
Domain architectures and catalytic mechanisms of typical non-ribosomal peptide synthase **(A)** and ε-PL synthetase (in *S.albulus*, *E. festucae* and *C. variabile*) **(B)** (Yamanaka et al., 2008; Xu et al., 2016; Purev et al., 2020; Jiang et al., 2021).

ε-PL synthetase (Pls, encoded by *pls* gene) has been characterized as a membrane-bounded non-ribosomal peptide synthase (NRPS)-like enzyme which catalyzes the polymerization of L-lysine monomers into ε-PL (Yamanaka et al., 2008). This enzyme has a typical NRPS adenylation domain (A domain) for the activation of substrate to aminoacyl-O-AMP, and a thiolation domain (T domain) that mediates localization and transfer of the activated substrate. However, the N-terminus of Pls carries neither a condensation domain (C domain) nor a thioesterase domain (TE domain), as found in typical NRPSs, but instead harbors three tandem domains (C1-C3) related to acetyltransferase functions and six transmembrane domains (TM1-TM6) (Figure 2) (Kito et al., 2013; Jiang et al., 2021). As mentioned above, Pls have been identified in some Ascomycota fungi and Coryneform actinobacteria. Interestingly, despite the low amino acid similarity (∼50%) between the amino acid sequences of *Strepromyces albulus* Pls and *E. festucae* Pls, or *S. albulus* Pls and *C. variabile* Pls, these three enzymes share similar domain architectures (Purev et al., 2020; Jiang et al., 2021). Beyond this, study also revealed that the acidic pH conditions in the ε-PL fermentation process facilitate the accumulation of intracellular ATP and Pls-mediated activation of substrate to aminoacyl-O-AMP, which are essential to the biosynthesis of ε-PL (Yamanaka et al., 2010).

As is well-known, ɛ-PL can be synthesized by Pls at pH 4.0, while hydrolyzed by ε-PL degradation enzyme (Pld) with the increasing pH at the end of the fermentation process (Kahar et al., 2001). The biological function of Pld is to maintain normal growth of the strain under the pressure of ε-PL by reducing its toxicity for the strain itself (Kito et al., 2002; Hamano et al., 2006; Yamanaka et al., 2010). Many efforts have been made to determine whether Pld participates in the regulation of ε-PL synthesis and control of polymer length. *In vitro* assays have shown that the incubation of Pls with ATP and L-lysine directly produced ɛ-PL of 3–17 L-lysine units, suggesting that biosynthesis and chain length of ε-PL can be directly controlled by Pls (Yamanaka et al., 2008). At present, two types of ɛ-PL degradation enzymes, named Pld and PldII, have been identified in ε-PL-producing *Streptomyces*, with exo-type and endo-type peptidase activities, respectively (Yamanaka et al., 2010). Theoretically, deletion of these two enzymes should reduce the loss of ɛ-PL and improve its overall titer during the fermentation. However, the inactivation of Pld and PldII showed no effect on the production or polymerization degree of ε-PL, which suggests that the biosynthesis and polymer length of ε-PL appears to be directly controlled by Pls, rather than Plds (Yamanaka et al., 2010).

### Factors Affecting ε-PL Molecular Weight During the Biosynthesis

In general, the biofunctionalization and practical application of biopolymers are tightly associated with its molecular weight (Sha et al., 2019). For instance, low molecular weight (<400 kDa) poly-gamma-glutamic acid (γ-PGA) is more functional than high molecular weight (>1000 kDa) for some bioengineering applications, such as a crop cryoprotectant or synergist for fertilizers (2 kDa), calcium absorption enhancer (11 kDa), and drug carrier (45–60 kDa) (Lee et al., 2018). Molecular weight is a main factor affecting the antimicrobial activity of ɛ-PL. However, the bitter flavor of commercial ε-PL products (3.2–4.5 kDa) limit their application in the food industry. This problem could be potentially resolved by appropriately reducing the chain length of the ε-PL polymers. Hence, understanding the key factors that affect ε-PL molecular weight can open new avenues for optimizing ε-PL biosynthesis, as well as for engineering the targeted synthesis of novel ɛ-PLs with desired chain lengths.

Polydispersity of ε-PL molecular weight is primarily determined by the ε-PL-producing strain (Takehara et al., 2010). The majority of obtained ε-PL polymers have nearly the same molecular weight as the first ones that were discovered (3.2–4.5 kDa), though some are much lower (Xu et al., 2019). Hirohara et al. (2007) found some actinomycetes produced ɛ-PLs with molecular weights of 4.1, 3.6, 3.2, 2.4, and 2.0 kDa. Notably, *Kitasatospora* sp. PL6-3 secretes an ε-PL with the highest reported molecular weight (5.01 kDa), although ɛ-PL production is poor in this strain (Ouyang et al., 2006).

As mentioned above, Pls is a main factor determining the degree of ε-PL polymerization (Yamanaka et al., 2010). Thus, one study used a “gene swap strategy” to integrate a *pls* gene from a short-chain ε-PL producer *Kitasatospora aureofaciens* into the long-chain ε-PL-high-yielding *S. albulus*, which resulted in high-yield synthesis of short-chain ε-PL (Xu et al., 2019). To further identify which functional domain determines ε-PL chain length, Hamano et al. (2014) carried out a random mutagenesis screen of C-terminal Pls domains by targeted error-prone PCR. The results showed that mutations (Pls-L883P and Pls-W646S) in the linker regions that connect the TM (1 and 2) and TM (3 and 4) domains leads to shorter chain length (from 21–32 to 10–23-mer and 9–23-mer, respectively). Saturation mutation of W646 and L883 further obtained a series of enzyme variants that produced short-chain ε-PL. Interestingly, no mutation in Pls was found to induce longer length in ε-PL chains (Hamano et al., 2014).

Intracellular microenvironments can affect the synthesis of metabolites and their associated primary and secondary metabolic pathways. It warrants mention that the polymerization degree of ε-PL is affected by the microenvironment of the host cell. Polymerization degree of ε-PL produced by *S. albulus* NK660 was found to be lower than that produced by NBRC14147, despite their respective *pls* genes sharing more than 99% similarity (Geng et al., 2014). *E. festucae* E437 is a novel ε-PL-producing strain that can reportedly produce 10.4 mg/L ε-PL with 28–34 L-lysine residues. Overexpression of the fungal Pls (from *E. festucae* E437) in *E. festucae* F11 (which produces negligible ε-PL) resulted in a lower degree of ε-PL polymerization than that in E437, suggesting that host cell have an effect on the degree of polymerization (Purev et al., 2020).

The addition of short-chain alcohols, including butanol, glycol, propanediol, or butanediol can also affect ε-PL molecular weight (Chen et al., 2018; Ding et al., 2018). Nishikawa and Ogawa (2002) were the first to find an unknown ε-PL derivative when glycerol was used as a carbon source in the fermentation. This ε-PL derivative was then identified as an ester formed between the terminal carboxyl group of ε-PL and the hydroxyl group of glycerol. These ε-PL-polyol esters were formed during the L-lysine polymerization process, rather than through an exogenous esterification reaction. Moreover, the quantity of L-lysine residues in the ε-PL ester decreased commensurately with increasing polyol concentration. Further research revealed that the addition of polyol lowered the ε-PL molecular weight through a terminal carboxyl esterification reaction between ε-PL and polyol which inhibits ε-PL elongation (Nishikawa and Ogawa, 2006). Glycerol is widely used as a carbon source for producing ε-PL, instead of glucose, which results in lower polymerization and higher productivity (Chen and Mao, 2013; Chen et al., 2018). Chemical modification of cyclodextrin, e.g., with sulfate, can further enhance the effects of glycerol, reducing the ε-PL molecular weight from 3.5 to 4.5 kDa to less than 2.5 kDa (Nishikawa, 2009).

In light of these studies, the “gene swap strategy” and the addition of short-chain alcohols both appear to be effective methods for increasing the production of ε-PL with lower molecular weight.

## Strain Improvement

To promote the industrial adoption of ɛ-PL, many lines of research have focused on engineering strains with elevated capacity for ɛ-PL synthesis. Table 1 summarizes studies of cell engineering strategies and the corresponding levels of ɛ-PL obtained with different ɛ-PL producers, and the detailed information is discussed below.

**TABLE 1:** Summary of the cell engineering strategies on ɛ-PL production.

Strategy	Microorganism			Fermentation mode	Substrate^a^	Detail	Result	Reference
**Mutagenesis, Genome shuffling and Ribosome engineering**								
Mutation by L-lysine analog AEC		*S. albulus* 11011A	120 h fed-batch fermentation		Glucose yeast extract	S-(2-aminoethyl)-L-cysteine (AEC)	5.7 g/L	Hiraki et al. (1998)
ARTP mutagenesis		*S. albulus* SAR 14–116	Shake flask fermentation		Glucose yeast extract	Atmospheric room temperature plasma (ARTP)	1.1 g/L	Xiang et al. (2020)
ARTP mutagenesis and Str resistance		*S. albulus* AS3-14	192 h fed-batch fermentation		Glycerol yeast extract	Streptomycin (Str)	41.2 g/L	Wang et al. (2015a)
Genome shuffling and glucose tolerance		*S. albulus* F3-4	145 h fed-batch fermentation		Glucose yeast extract	High glucose concentration	13.5 g/L	Li et al. (2012)
Genome shuffling interspecific hybridization		*S. albulus* FEEL-1	168 h fed-batch fermentation		Glucose yeast extract	Interspecific hybridization of five *Streptomyces* sp	24.5 g/L	Li et al. (2013)
Interspecific hybridization		*S. albulus* LS-84	196 h fed-batch fermentation		Glucose yeast extract	Interspecific hybridization between *S. albulus* and *B.subtilis*	32.6 g/L	Li et al. (2018)
Reducing self-inhibition for increase of ɛ-PL production		*S. albulus* F4-22	173 h fed-batch fermentation		Glycerol beef extract	High ɛ-PL tolerance	39.9 g/L	Zhou et al. (2015)
Genome shuffling and ribosome engineering		*S. albulus* AG3-28	240 h fed-batch fermentation		Glucose yeast extract	Improve gentamycin resistance of by genome shuffling	56.5 g/L	Wang et al. (2016)
Str-resistant mutation		*S. albulus* SS-62	Shake flask fermentation		Glucose yeast extract	Continuous introduction of Str resistance	3.04 g/L	Liu et al. (2019b)
Double antibiotic resistant mutations (Str, Gen)		*S. albulus* SG-31	174 h fed-batch fermentation		Glucose yeast extract	Str, gentamycin (Gen)	57.6 g/L	Wang L. et al. (2017)
Multiple antibiotic (Str, Gen, Rif, Gnt, Par, Lin) resistant and pH shock strategy		*S. albulus* R6	192 h fed-batch fermentation		Glucose yeast extract	Str, Gen, Rifamycin (Rif), geneticin (Gnt), paromomycin (Par), lincomycin (Lin)	70.3 g/L	Wang et al. (2019)
**Gene engineering**							
Overexpression gene for ɛ-PL biosynthesis and addition of citrate		*S. albulus* Q-PL2	72 h fed-batch fermentation		Glucose yeast extract	Overexpression of ɛ-PL synthetase gene (*pls*)*,* addition of 2 g/L sodium citrate	20.1 g/L	Wang C. et al. (2020)
Overexpression gene for ɛ-PL biosynthesis		*E. festucae* Ptef::*Epls*	Shake flask fermentation		Potato glucose	Overexpression of fugal ɛ-PL synthetase gene (*Epls*)	69.9 mg/L	Purev et al. (2020)
Overexpression of a transcription factor to activate ɛ-PL synthesis		*E. festucae* Ptef::*vibA*	Shake flask fermentation		Potato glucose	Overexpression of *vibA* (a gene of transcription factor)	18.6 mg/L	Purev et al. (2020)
Heterologous expression of ɛ-PL synthetase in *Streptomyces lividans* ZX7		*S. lividans* ZX7-*pls*	120 h fed-batch fermentation		Glucose yeast extract	The ɛ-PL synthetase is cloned from *S. albulus* NK660	2.5 mg/L	Geng et al. (2014)
ɛ-PL synthetase swap strategy		*S. albulus* PD-5	168 h fed-batch fermentation		Glucose yeast extract	Knock out the *plsI* gene and express *plsII* gene with native promoter in *S. albulus* PD-1	23.6 g/L	Xu et al. (2019)
Increase the expression efficiency of ribosome		*S. albulus* M-Z18-*frr*	25 h batch fermentation		Glucose yeast extract	Overexpression of *frr* (a gene for ribosome recycling factor)	3.7 g/L	Liu et al. (2019a)
Removal of feedback inhibition in L-lysine biosynthesis pathway		*S. albulus* CR1-*ask* (M68V)	168 h fed-batch fermentation		Glucose yeast extract	Express the site-mutated aspartokinase (encoded by *ask* gene)	15 g/L	Hamano et al. (2007)
Enhancement of L-lysine biosynthesis pathway		*S. diastatochromogenes* 2#-2	168 h fed-batch fermentation		Glucose yeast extract	Express the dihydrodipicolinate synthase gene (*dapA*)	30.54 g/L	Li et al. (2021)
Inactivation of the by-product genes		*S. albulus* NBRC14147	30 h batch fermentation		Glucose yeast extract	Inactivation of *ttm* and *nys* clusters	3.5 g/L	Yamanaka et al. (2020)
Alleviating oxygen limitation in ɛ-PL synthesis		*S. albulus* PD-2	168 h fed-batch fermentation		Glucose yeast extract	Overexpressing *Vitreoscilla* Hemoglobin gene (*vgb*)	34.2 g/L	Xu et al. (2015b)
Enhancement of nitrogen translocation and utilization efficiency		*S. albulus* PD-1-*amtB*	168 h fed-batch fermentation		Glucose yeast extract	Overexpressing the ammonium transporter gene (*amtB*)	35.7 g/L	Xu D. et al. (2018)

a Represent the carbon/organic nitrogen resource in medium.

### Mutagenesis, Genome Shuffling and Ribosome Engineering

A strong ɛ-PL producer is the foundation of any commercially successful fermentation system, which can overcome the extremely slow growth rate and low ɛ-PL production of the wild-type strain. Due to the lack of effective genetic transformation methods for these recalcitrant bacterial strains, conventional mutagenesis served as the primary method for engineering new strains in the three decades following the initial discovery of ɛ-PL-producing strains (Shima and Sakai, 1977; Hamano et al., 2005). Since excess accumulation of L-lysine and L-glycine inhibit the activity of aspartokinase (Ask) in the L-lysine synthetic pathway, mutagenesis studies combined with screening for the “L-lysine analog (S-(2-aminoethyl)-L-cysteine (AEC))+glycine-resistant” mutants ultimately resulted in a mutant strain that could secrete 4-fold higher ɛ-PL than wild type (Hiraki et al., 1998). Currently, the utilization of atmospheric and room temperature plasma (ARTP) mutagenesis results in 18.46% improvement in ɛ-PL production, which may be attributable to an up-regulation in metabolite levels necessary for L-lysine synthesis and degradation pathways, based on metabolomic profiles (Xiang et al., 2020).

Carbon flux from substrate is another limiting factor for production. To increase substrate utilization, Li et al. (2012) used genome shuffling to obtain a recombinant strain with high glucose tolerance. Subsequently, genome shuffling was combined with interspecific hybridization of five ɛ-PL-producing *Streptomyces* to further improve the metabolic flow of the glycolysis pathway and TCA cycle, resulting in a strain that could produce 24.5 g/L ɛ-PL (Li et al., 2013). More recently, similar work facilitated the integration of traits from *S. albulus* and *B. subtilis* enabling ɛ-PL production 256.1% higher than that of the parent strain (Li et al., 2018). Additionally, since the auto-inhibition caused by the accumulation of ɛ-PL may limit cell growth and ɛ-PL biosynthesis, genome shuffling has been also used to rapidly increase the ɛ-PL tolerance and production (Zhou et al., 2015).

Another highly noteworthy strategy for semi-rational screening of high ɛ-PL producers is ribosome engineering. It has been reported that the introduction of resistance to some macrolide antibiotics can strongly activate the production of various secondary metabolites, including actinorhodin and salinomycin (Ochi, 2007; Zhang et al., 2019). Through this technique, mutations conferring resistance to streptomycin and five other antibiotics were successively induced in *S. albulus* to generate the *S. albulus* R6 mutant strain, which exhibited the highest reported ɛ-PL production of 70.3 g/L, 2.79-fold greater than the original strain, using an optimized acidic pH shock strategy (Wang et al., 2019). Mutations in protein S12 (Q856H) and RNA polymerase (R99P) of the R6 strain could be responsible for the increased transcription of the *pls* gene and elevated activities of key enzymes in ɛ-PL biosynthetic pathway, consequently increasing the level of Pls activity, as well as L-lysine and ATP concentrations available for ɛ-PL production (Wang et al., 2019). To explore the mechanism by which streptomycin resistance leads to higher productions, a high ɛ-PL-producing mutant was generated by continuous selection for streptomycin resistance (and hence increasing mutations) (Liu et al., 2019b). Comparative proteomics then revealed that up-regulated protein expression related to L-lysine metabolism, energy metabolism, transcriptional regulation and translation pathways together facilitated an intracellular metabolic environment conducive to secretion of ɛ-PL. Although ribosome engineering has rapidly and effectively enhanced ɛ-PL production, the regulatory relationship between the mutated ribosome and RNA polymerase and ɛ-PL metabolism remain unclear. Currently, the application of a high-throughput screen, or a combination of ribosome engineering with conventional screening and genome shuffling can together enable further improvement to the efficiency of generating high ɛ-PL-producing strains (Wang et al., 2016; Liu et al., 2017; Wang L. et al., 2017). However, these processes are time-consuming and labor-intensive, and it remains difficult to reroute metabolic flux towards ɛ-PL production through well-established methods.

### Gene Engineering

**FIGURE 3:**
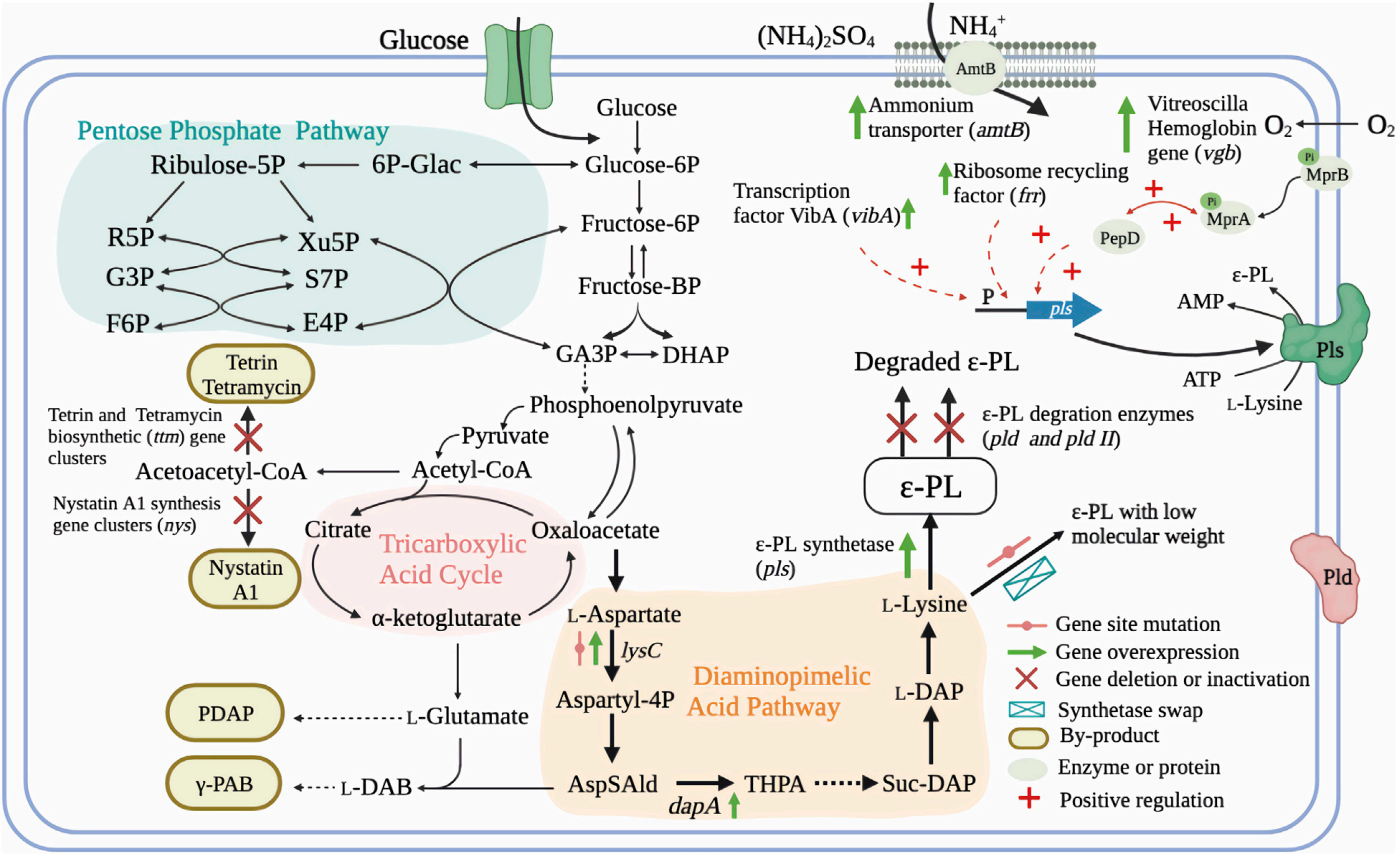
Genetic engineering strategies for the regulation of ɛ-PL production and molecular weight and in ɛ-PL-producing *Streptomyces* and *endophytic fungi* (Hamano et al., 2007; Takehara et al., 2008; Yamanaka et al., 2010; Xia et al., 2013; Hamano et al., 2014; Xu et al., 2015a; Xu T. et al., 2018; Liu et al., 2019a; Pan et al., 2019a; Xu et al., 2019; Purev et al., 2020; Wang A. et al., 2020; Yamanaka et al., 2020; Li et al., 2021). AmtB, ammonium transporter; MprA/B and PepD, a signal transduction system; GA3P, glyceratldehyde-3-phosphate; DHAP, dihydroxy acetone phosphate; L-DAB, L-diaminobutanoic acid; γ-PAB, γ -poly-L-diaminobutanoic acid; PDAP, poly-L-diaminopropionic acid; AspSAld, L-Asparte-Semialdehyde; THPA, Hydroxy-tetrahydrodipicolinate; Suc-DAP, Succinyl-diaminoheptanedioate; L-DAP, L-diaminoheptanedioate; ɛ-PL, ε-poly-L-lysine.

Genetic information provided by whole genome sequencing can provide a comprehensive picture of intracellular physiology and metabolism. Comparative genome analysis, as well as proteomic and metabolomic approaches can also guide directed evolution of strains through rational genetic engineering (Wang et al., 2015b; Xiang et al., 2020). For example, genome analysis of fully sequenced ɛ-PL-producing *S. albulus* ZPM revealed the presence of more than 40 secondary metabolite biosynthetic gene clusters, approximately half of which were polyketide synthases (PKSs) or non-ribosomal peptide synthetases (NRPSs) (Wang et al., 2015b). This finding indicates the main challenges related to ɛ-PL production, i.e., low yield and low production, respectively arising due to co-products potentially generated by these biosynthetic gene clusters during fermentation (Figure 3), and unknown rate-limiting steps in the ɛ-PL biosynthetic pathway (Yamanaka et al., 2020). These issues together limit the application of genetic and metabolic engineering in ɛ-PL-producing strains.

One commonly applied strategy for enhancing poly(amino acid) production such as γ-PGA and ɛ-PL involves increasing the intracellular availability of precursors (Cao et al., 2018). To construct a high-yield ɛ-PL producer, the first gene delivery system was developed for *S. albulus* (Hamano et al., 2005). Since wild-type Ask in *S. albulus* is subject to feedback inhibition by L-lysine and L-threonine, Ask was mutated (M68V) to eliminate this inhibitory effect and then homologously expressed in *S. albulus*, which showed increased biosynthesis of ɛ-PL (Hamano et al., 2007). More recently, dihydrodipicolinate synthase (DapA), a key enzyme in L-lysine biosynthesis pathway, has also been overexpressed for enhanced ɛ-PL production, which contributed to a 19.8% increase in ɛ-PL production by *S. diastatochromogenes* (Li et al., 2021).

L-lysine is the sole precursor of ɛ-PL, which is polymerized by membrane-associated Pls. Hence, enhancing Pls expression represents a viable strategy for increasing ɛ-PL productivity. One previous study showed that overexpression of the *pls* gene using the kasOp* promoter and ribosome binding site from phage Φ C31 capsid protein in a modified *S. albulus* strain resulted in 88.2% higher ɛ-PL production than that of wild type. Consequently, this strain achieved 20.1 g/L ɛ -PL in a 3 days fermentation (Wang A. et al., 2020). Similar results were observed in the endophytic fungus *E. festucae* E437 overexpressing the *Epls* (fungal ε-PL synthetase) gene, for which ɛ-PL production was enhanced 6.72-fold over that of wild type (Purev et al., 2020).

Since ε-PL is a secondary metabolite, its production is strictly regulated by intracellular regulatory mechanisms, as with other secondary metabolites. For instance, *E. festucae* F11 carries all of the genes necessary for synthesis of ε-PL, but these genes are not all transcribed and almost no ɛ-PL is produced. Introduction of the VibA transcription factor into *E. festucae* F11 led to induction of Pls activity and accumulation of ɛ-PL. Furthermore, overexpression of VibA in another *E. festucae* (E437) also led to a 3.7-fold increase in ε-PL production (Purev et al., 2020). Similarly, HrdD, a sigma factor mediating sensitive responses to pH change, was found to bind the *pls* gene promoter regions, suggesting the potential for pH-related regulation. A mutation in HrdD discovered through genomic comparisons between high- and low-yield strains provided a potential target site for engineering improved strains (Wang et al., 2015b). Moreover, proteomic analysis revealed that ribosome recycling factor is related to elevated expression of *pls* gene and ɛ-PL productivity in a hyper-yielding *Streptomyces* (Liu et al., 2019a). Transcriptome analysis of the acid tolerance response by *S. albulus* M-Z18 uncovered a signal transduction system (MprA/B and PepD) that can positively regulate the *pls* transcription, and thus could significantly enhance the production of ɛ-PL (Figure 3) (Pan et al., 2019a; Wang C. et al., 2020).

Elimination of metabolic pathways that divert a precursor or metabolite from the target molecule into unexpected byproducts, has often been reported to increase the concentration of target products. Most byproducts found in ɛ-PL-producing *Streptomyces* species were biopolymers or antibiotic compounds, such as poly(gamma-L-diaminobutanoic acid) (γ-PAB), γ-PGA, poly(L-diaminopropionic acid) (PDAP), and tetramycin A and B, among others (Takehara et al., 2008; Nishikawa and Kobayashi, 2009; Xia et al., 2013; Yamanaka et al., 2020). Interestingly, studies reporting low co-production of γ-PAB and PDAP with ɛ-PL, did not block or suppress these pathways in their efforts to increase ɛ-PL production. Recently, Yamanaka and colleagues revealed that genes for biosynthesis of five polyene macrolide antibiotics (tetramycin A and B, tetrin A and B, and a trace amount of nystatin A1) are also present in the genome of ɛ-PL-producing strain *S. albulus* 14147. Targeted inactivation of the four other antibiotics in a nystatin A1-deficient mutant led to enhanced ɛ-PL biosynthetic flux and an approximate 20% increase in ɛ-PL production (Yamanaka et al., 2020).

As a secondary metabolite, any enhancement to metabolic rates in the producer cell has the potential to improve ɛ-PL biosynthesis along with other metabolites. To this end, cellular metabolic activity and microbial biomass can be promoted in multiple ways. For instance, oxygen is a limiting factor for aerobic metabolism in submerged liquid fermentations, especially under high cell density. Oxygen supply is a well-known bottleneck in ɛ-PL fermentation because the increased ɛ-PL concentration and cell density result in heavy viscosity of the culture broth, consequently limiting oxygen transfer from the sterile gas to the growth medium. *Vitreoscilla* Hemoglobin (VHb, encoded by the *vgb* gene) can bind with oxygen at low concentrations and transport it to sites in the cell where greater oxygen supply is needed for metabolic processes. Expression of VHb has been historically used to enhance metabolic efficiency and biomass of microbial cells. For example, Xu et al. (2015b) introduced the *vgb* gene (GenBank No. M30794.1) into *S. albulus* PD-1, the mutant *S. albulus* PD-2 (containing pIB 139-*vgb*) presented an improved oxygen uptake rate, ATP levels, and transcription of the *pls* gene, which resulted in a 50.7% increase in ɛ-PL production compared to the wild type. Similarly, Gu et al. (2016) overexpressed *vgb* in *S. albulus* NK660, thereby leading to 26.7 and 14.6% increases in ɛ-PL production and cell dry weight, respectively, over that of wild type.

In addition to oxygen requirements, high nitrogen availability is also essential for L-lysine synthesis, since each L-lysine has two nitrogen atoms while most other amino acids contain only one nitrogen atom. To improve nitrogen translocation and utilization efficiency, the ammonium transporter gene *amtB* was overexpressed in *S. albulus* PD-1-*amtB*, resulting in an increase ε-PL production of 57.2% compared to the wild type (Xu D. et al., 2018). S-adenosylmethionine synthetase (SAM, encoded by the *metK* gene) is a methyl donor that provides a methyl group to several reactions necessary for primary and secondary metabolism. However, an *S. albulus* NK660 *metK*-overexpressing strain showed no discernible increase in ε-PL but had 9.79% more biomass than wild-type NK660 (Gu et al., 2016). Therefore, the combination of this strategy with other approach may redirect metabolic flux from cell growth to the biosynthesis of ε-PL. Collectively, although the transformation efficiency for gene editing of ε-PL-producing Streptomycete and endophytic fungi is still low, redirecting the metabolic pathways towards ε-PL biosynthesis has gradually become more feasible using genetic or metabolic engineering.

## Process Engineering

### Optimizing Environmental Conditions

In ɛ-PL fermentation, culture pH naturally decreases to about 3.0 if acidity is not controlled. In this case, the metabolism of ɛ-PL-producing strains can be seriously inhibited by fluctuations in the intracellular microenvironment, resulting in the low biomass and ɛ-PL production. Therefore, pH-control strategies through automatic feeding of an alkali solution (i.e., NaOH or NH_4_OH) is a reliable method for ensuring sufficient accumulation of high biomass. Kahar et al. (2001) found that pH 4.0 is optimal for ε-PL formation, while higher pH (>4.5) is better for cell growth. Therefore, a strategy to control pH was clearly advantageous in which pH was first maintained higher than 5.0 to increase biomass, then pH was allowed to naturally decrease to 4.0, where it was then maintained for accumulation of ɛ-PL. Under these conditions, ε-PL production was enhanced from 5.7 to 48.3 g/L. Through an analogous pH control strategy, Shih and Shen (2006) raised the pH for cell growth to 6.8 and observed a 258% increase in ε-PL production.

**FIGURE 4:**
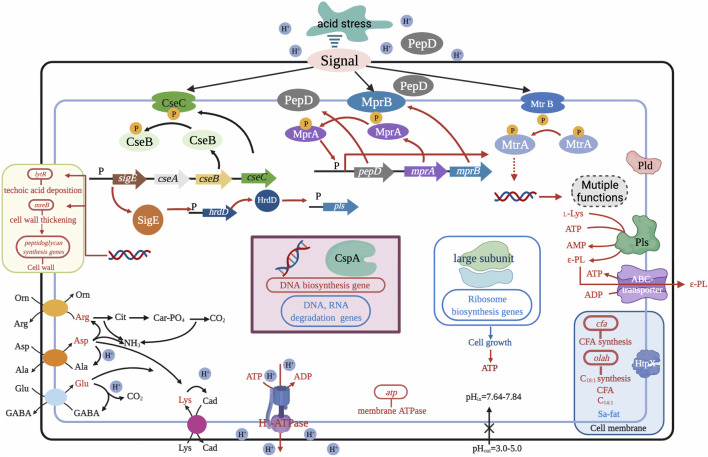
Possible mechanism for the acid tolerance response by *S. albulus* M-Z18. Figure modified from Wang C. et al. (2020). Arg, arginine; Asp, aspartate; L-Lys, L-lysine; C_18:1_, oleic acid; Orn, ornithine; Ala, alanine; Cad, cadaverine; C_14:1_, myristoleic acid; Cit, citrulline; Glu, glutamate; ε-PL, ε-Poly-L-lysine; Sa-fat, saturated fatty acid; Car-PO_4_, carbamoyl phosphate; GABA, γ-aminobutyrate; CFA, cyclopropane fatty acid.

Recent research has shown that temporary (5–12 h) suppression of low pH in the exponential phase of *S. albulus* culture can contribute to higher cell viability and capacity for ε-PL synthesis. A two-stage pH control strategy was first proposed by Chen X. S. et al. (2011), with pH shifting from 3.5 to 3.8 at 36 h for optimal rates of ɛ-PL formation during the exponential and stationary phases, which ultimately tripled ε-PL production from 9.13 to 30.11 g/L. Based on this method, Ren et al. (2015c) divided the ε-PL fermentation into three stages and developed an acid pH-shock (PS) strategy, including *1*) enrichment and cell culture at pH 5.0; *2*) activation of cell metabolic activity at pH 3.0; and *3*) secretion of ε-PL at pH 4.0. ε-PL production and accumulation rates were 54.7 and 6.84 g/L/day using this method, 52.50% higher than those of control (no pH shock). In addition to exceptional ε-PL production, biomass, mycelial viability, and intracellular ATP concentration were all significantly higher under PS condition than those of the control (Ren et al., 2015b). The underlying relationship between high ε-PL production, biomass, mycelial viability, ATP accumulation, and pH shock was further explained by transcriptomic profiling at the early, middle, and late stages of ε-PL fermentation, which showed up-regulation of transcriptional regulatory genes involved in nitrogen metabolism, energy metabolism, electron respiratory chain, and redox homeostasis (Pan et al., 2019a). Other work has shown that the MprA/B and PepD signal transduction system, which positively regulate the transcription and expression of *pls* gene, are also activated by pH shock. Deletion of *mprA/B* and *pepD* genes resulted in 83.5–93.2% and 52.1–60.6% respective reductions in *pls* transcription and ε-PL production (Pan et al., 2019b). Acid tolerance response by *S. albulus* has also been considered a contributing factor in high ε-PL production. Pan et al. (2017) found that induction of pH stress (pH 3.0) in the PS strategy enhanced acid tolerance by *S. albulus*. Transcriptomic analysis at different environmental pH levels (3.0, 4.0, and 5.0) further revealed that pH stress induced an overall acid tolerance response by *S. albulus* M-Z18, while concomitant ε-PL production was proposed to be a response mechanism to combat acid stress (Figure 4) (Wang A. et al., 2020).

Dissolved oxygen (DO) is also an important parameter in the ε-PL fermentation process for its effects on ε-PL biosynthesis, mycelial biomass, and substrate utilization rates. However, oxygen supply is often limited in ε-PL fermentation broth because of high oxygen consumption and cell density. Bankar and Singhal (2011) proposed a two-stage DO control strategy for increased biomass and ε-PL production in *Streptomyces noursei* NRRL 5126 through investigation of growth kinetics under different aeration, agitation, and DO levels. The most appropriate DO levels during the production phase differed depending on the desired products. An interesting strategy was then developed for co-producing PDAP and ε-PL by controlling pH and DO concentrations. In this strategy, pH control is similar to that of Kahar’s strategy, while DO was successively maintained at 15 and 30% for PDAP and ε-PL production, respectively. This method led to an increased yield of 21.8%, which is the highest reported yield among similar production systems (Xu et al., 2015c). However, the high agitation speeds and aeration require high power consumption and lead to undesirable impacts on mycelial morphology and ε-PL production.

Another method to solve the problem of oxygen limitation is the addition of an oxygen-vector. Xu et al. (2015a) improved oxygen supply and maintained DO concentration at >32% saturation by adding 0.5% n-dodecane in broth. The enhanced ε-PL and biomass concentrations (by 31.6 and 20.7%, respectively) under conditions of elevated oxygen were likely related to stronger carbon and energy metabolism. In addition, *Vitreoscilla* hemoglobin, mentioned above, can also help to enhance respiration and oxidative phosphorylation by binding and delivering the oxygen to terminal respiratory oxidase (Xu et al., 2015b; Gu et al., 2016).

Additionally, some novel strategies have also been proposed for increasing ε-PL production. Notably, *in situ* product removal (ISPR) is an integrated method that combines production and separation for avoiding end-product toxicity/feedback inhibition, thereby simultaneously enhancing the overall process efficiency. Liu et al. (2011) selected ion-exchange D152 resin as an effective adsorbent to develop a resin-based ISPR system, ultimately obtaining 3.62- and 6.22-fold increases in ε-PL production in shake-flask and 5-L fermenter, respectively. In other research, ISPR was combined with a cell immobilization technique using a loofah sponge as an immobilization carrier. Through this combination method, immobilized cells of *S. ahygroscopicus* GIM8 could be repeatedly used, resulting in a 10-fold increase in ε-PL production, from 0.82 to 8.05 g/L (Liu et al., 2012b).

### Optimizing Nutrient Supply

**FIGURE 5:**
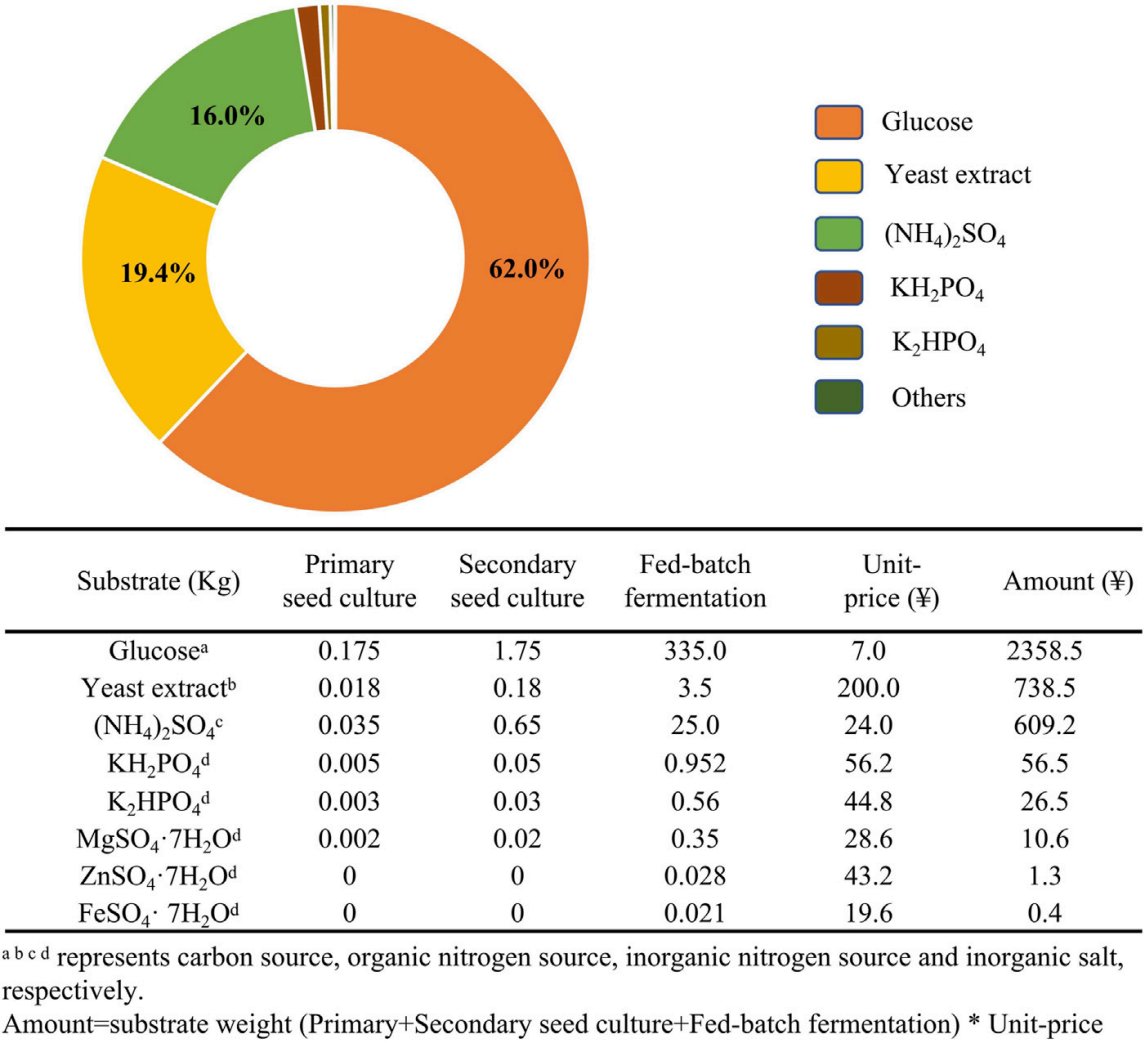
Substrate costs for a *Streptomyces* sp. fermentation in a 1-t bioreactor with M3G medium.

Media composition can directly affect cell growth and biosynthesis by microorganisms. M3G medium (Figure 5) was the first reported medium for ε-PL fermentation and remains commonly used. Various approaches for optimizing medium have been designed based on this original culture medium, such as the single-factor method, orthogonal array method, response surface methodology (RSM), and artificial neural networks (ANN). These methods have facilitated significant (56.4–200%) improvements to ε-PL production (Chheda A. H. and Vernekar M. R., 2015; Bhattacharya et al., 2017; Guo et al., 2018). Among these studies, Plackett-Burman assays showed that sources of carbon (typically glucose and glycerol) and organic/inorganic nitrogen (i.e., yeast extract, peptone, and (NH_4_)_2_SO_4_) were major influencing factors in ε-PL production (Chen X. S. et al., 2011; Guo et al., 2018).

Providing nutrients that participate in the ε-PL metabolism is another effective method for enhancing ε-PL production. Chen et al. (2013) improved ε-PL production by 6.2% through the addition of L-lysine to broth, and determined that the addition of L-lysine directly contributed to ε-PL biosynthesis by L-[U-^13^C] lysine labeling. Similarly, Liu et al. (2012a) discovered that the addition of L-lysine or D-lysine could lead to greater ε-PL production and biomass, although these two amino acids participated in different metabolic pathways in the ε-PL-producing strain. Citric acid is an important TCA cycle intermediate, and the addition of citric acid strengthened ε-PL synthesis while reducing the PDAP by-product. The regulatory mechanism underlying this effect is potentially due to citric acid inhibition of the TCA cycle, which redirects the metabolic flux into the L-aspartate biosynthetic pathway, thereby increasing the ε-PL production from 21.7 to 29.7 g/L (Xia et al., 2014a). Based on the above evidence, feeding L-aspartate coupled with citric acid could further lead to higher productions in *B. cereus* and *S. noursei* by 6-fold and 5.5-fold, respectively (Chheda A. H. and Vernekar M. R., 2015; Chheda A. H. and Vernekar M. R., 2015). Analogously, supplementation with various nitrogen-rich feedstocks, such as beef extract, soybean powder, yeast extract, fish meal, and corn steep liquor can provide abundant L-aspartate, L-glutamate, L-arginine, and L-lysine, which can remarkably improve rates of cell growth and ε-PL synthesis (Liu et al., 2015). Iron, manganese, and cobalt are also reportedly involved in the expression of *pls* genes. Kobayashi and Nishikawa (2007) added the above metals in liquid culture medium, which enhanced the production of ε-PL by *Kitasatospora kifunense*.

### Low-cost Agri-Industrial Wastes Fermentation

The price of medium represents a substantial proportion of the operating costs in ε-PL industrial fermentations. In 192 h *S. albulus* fermentation in a 50-L bioreactor, the cost of the carbon source (glucose) accounts for 62.0% the total substrate costs for M3G medium (Figure 5). Thus, lower cost agri-industrial waste products, such as crude glycerol and sugar cane have been tested as attractive potential alternative carbon sources for ε-PL fermentation.

*Streptomyces* dissimilates glycerol (a waste product of biodiesel production) *via* the phosphorylative pathway. In *S. albulus*, glycerol is converted into glycerol-3-phosphate by glycerol kinase (GK), then oxidized into dihydroxyacetone phosphate (DHAP) by glycerol-3-phosphate dehydrogenase (G-3-PDH), which then directly participates in the glycolysis pathway or enters the gluconeogenesis pathway after it is isomerized to glyceraldehyde-3-phosphate. Previous studies have demonstrated that the rate of ε-PL formation by *S. albulus* cells grown on glycerol was 40% higher compared with cells grown on glucose. This result may be attributable to the 25% higher flux of glycerol into ε-PL synthesis compared to glucose (Chen and Mao, 2013). Glycerol-derived ε-PL has a characteristically lower chain length and toxicity for ε-PL-producing strains, which could at least partially explain the enhanced production of ε-PL (Ding et al., 2018). In addition, the activities of GK and G-3-PDH, key enzymes in glycerol assimilation pathway, may be inhibited by a limited oxygen supply in later fermentation stages (Du et al., 2021). Hence, tests of a glucose-glycerol mixed carbon source (MCS) for culture *S. albulus* resulted in a substantially higher growth rate and ε-PL productivity (Chen et al., 2012; Zeng et al., 2014). Transcriptional analyses further showed that glucose and glycerol could be simultaneously utilized in a MCS with no mutual transcriptional suppression, thus providing more carbon skeletons for glycolysis pathway (Zeng et al., 2017). The overall strengthened central carbon metabolism, amino acid metabolism, energy metabolism, and Pls activity could all contribute to the accumulation of higher biomass and ε-PL production (Zhang et al., 2018; Zeng et al., 2019). However, the rate of ε-PL biosynthesis with MCS declined sharply in the late phase of fed-batch fermentations. Cell physiology and transcriptomic analyses showed that excessive oxidative stress induced by reactive oxygen species (ROS) could contribute to the decreased capacity for ε-PL synthesis (Zeng et al., 2015). This problem could be partly solved by the addition of reducing agents, such as antioxidant glutathione and exogenous astaxanthin, which could ameliorate excessive oxidative stress and help to maintain a high ε-PL formation rate during the fermentation (Yan et al., 2018; Li et al., 2020).

As a by-product of the sugar industry, sugar cane has also been applied in ε-PL fermentation by *S. albulus* and *Bacillus sp.* (Shukla and Mishra, 2013; Xia et al., 2014b)*.* When pretreated cane molasses and hydrolysate of *Streptomyces* cells (HSC) were used as carbon and nitrogen sources, respectively, 20.6 g/L of ε-PL and 5.2 g/L of PDAP were obtained in 1-t fermenter (Xia et al., 2014b). Another method utilizing remnant glucose from levan production as an alternative carbon source produced 4.37 g/L of ε-PL, which is environmentally friendly and economical since it converts outdated feedstocks into high-value products (Shih et al., 2011).

The price of organic nitrogen sources (e.g., yeast and beef extracts) represents the second largest proportion (19.4%) of substrate costs. Corn powder (biowaste corncob residue), fish meal, and soybean powder are commonly used substitutes for organic nitrogen sources (Figure 5). Ren et al. (2015c) produced 54.7 g/L ε-PL by culturing *S. albulus* M-Z18 on glycerol and fish meal. These feedstocks combined with talc microparticles to alter mycelial morphology resulted in ε-PL production as high as 62.36 g/L, which is the highest reported production from agri-industrial wastes (Ren et al., 2015d). Recent work by Xu et al. (2017) in which a repeated-batch solid-state fermentation (SSF) was performed using a mixed carbon source of wheat bran (2:1, w/w), rapeseed cake, and glucose (4% w/w) showed a maximum ε-PL production of 86.62 mg/g substrate after eight repeated batches of SSF. Overall, several innovative strategies have been developed to increase ε-PL accumulation and reduce the cost of raw materials, with varying success (Table 2).

**TABLE 2:** Summary of the process engineering strategies on ɛ-PL production.

Strategy	Microorganism	Fermentation mode	Substrate^a^	Detail	Result	Reference
pH control strategy	*S. albulus* no.410	192 h fed-batch fermentation	Glucose yeast extract	Phase I: pH 5.0 for cell growth	48.3 g/L	Kahar et al. (2001)
				Phase II: pH 4.0 for ɛ-PL fermentation		
Novel two-stage pH control strategy	*S. albulus* M-Z18	192 h fed-batch fermentation	Glucose yeast extract	Phase I: pH 3.5	30.11 g/L	Chen X. S. et al. (2011)
				Phase II: pH 3.8 for optimal ε-PL formation rate		
pH-shock strategy	*S. albulus* M-Z18	192 h fed-batch fermentation	Glycerol fish meal corn steep liquor	Phase I: pH 5.0 for cell growth	54.7 g/L	Ren et al. (2015c)
				Phase II: pH naturally decrease to 3.0 for activation of cell metabolic activity		
				Phase III: pH 4.0 for secretion of ε-PL		
pH-shock and addition of microparticles	*S. albulus* M-Z18	192 h fed-batch fermentation	Glycerol fish meal corn steep liquor	Changing mycelia morphology for enhancement of ε-PL production by adding talc microparticles	62.36 g/L	Ren et al. (2015d)
DO control strategy	*S. noursei* NRRL 5126	144 h fed-batch fermentation	Glucose yeast extract	Phase I: constant DO at 40% in growth phase	2.0 g/L	Bankar and Singhal (2011)
				Phase II: 20% in production phase		
pH and DO control strategy	*S. albulus* PD-1	180 h fed-batch fermentation	Glucose yeast extract	Phase I: pH 5.0	PDAP: 9.6 g/L	Xu et al. (2015c)
				Phase II: pH 4.0, DO at 15% for PDAP production	ε-PL: 29.4 g/L	
				Phase III: pH 4.0, DO at 30% for ε-PL production		
Addition of oxygen-vectors to increase DO concentration	*S. albulus* PD-1	168 h fed-batch fermentation	Glucose yeast extract	Adding 0.5% n-dodecane	30.8 g/L	Xu et al. (2015a)
*In situ* product removal (ISPR) for reducing ε-PL toxicity	*S. ahygroscopicus* GIM8	192 h fed-batch fermentation	Glucose yeast extract	Add two bags of D152 resin as adsorbent of ε-PL	23.4 g/L	Liu et al. (2011)
ISPR and cell immobilization strategy	*S. ahygroscopicus* GIM8	192 h fed-batch fermentation	Loofah sponge	D152 resin as adsorbent	8.05 g/L	Liu et al. (2012b)
				Loofah sponge was used as immobilization carrier		
Ingle-factor method and orthogonal array method	*B. cereus*	Shake flask fermentation	Glucose yeast extract	Optimization of carbon resource, nitrogen resource, and salts in medium	83.49 mg/L	Chheda and Vernekar (2014)
Response surface methodology and Plackett Burman	*S. albulus* M-Z18	Shake flask fermentation	Glycerol beef extract	Studying the effect of glycerol, (NH_4_)_2_SO_4_ and K_2_HPO_4_ on ε-PL production	2.27 g/L	Chen, X. et al. (2011)
Artificial neural networks method	*B. licheniformis*	Shake-flask fermentation	Glucose yeast extract	Optimization of glucose, yeast extract, MgSO_4_ and FeSO_4_ by artificial neural networks method	3.56 g/L	Bhattacharya et al. (2017)
L-lysine addition	*S. albulus* M-Z18	168 h fed-batch fermentation	Glycerol yeast extract	Addition of 2 g/L L-lysine	37.6 g/L	Chen et al. (2013)
L-lysine and D-lysine addition	*S. ahygroscopicus* GIM8	Shake flask fermentation	Glucose yeast extract	Addition of 3 mM L-lysine	1.06 g/L	Liu et al. (2012a)
				Addition of 3 mM D-lysine	1.2 g/L	
Addition of citric acid	*S. albulus* PD-1	168 h fed-batch fermentation	Glucose yeast extract	Maintain the citric acid concentrations at 4 g/L	29.7 g/L	Xia et al. (2014a)
L-aspartic acid coupled with citric acid	*B. cereus*	120 h fed-batch fermentation	Glucose yeast extract	Adding 5 mM citric acid (24 h) and 2 mM L-aspartic acid (36 h)	0.56 g/L	Chheda and Vernekar (2015b)
Supplements of various nitrogen-rich nutrients	*S. ahygroscopicus* GIM8	288 h fed-batch fermentation	Glucose yeast extract	Optimized the time and addition concentration for organic nitrogen (yeast extract, soybean powder, and corn powder)	28.2 g/L	Liu et al. (2015)
Addition of iron	*K. kifunense*	Shake flask fermentation	Glycerol yeast extract	Study the effect of iron, manganese and cobalt on ɛ-PL synthesis	34.6 mg/L	Kobayashi and Nishikawa (2007)
MCS strategy	*S. albulus* M-Z18	174 h fed-batch fermentation	Glycerol glucose yeast extract	Glucose and glycerol as mixed carbon (MCS)	35.14 g/L	Chen et al. (2012)
Addition of antioxidant GSH	*Streptomyces* sp. AF3-44	168 h fed-batch fermentation	Glycerol yeast extract	Addition of 4.5 mM glutathione (GSH) to reduce oxidative stress	46.5 g/L	Yan et al. (2018)
Addition of antioxidant exogenous astaxanthin	*S. griseofuscus* LS-1	192 h fed-batch fermentation	Glycerol yeast extract	Addition of 1 g/L exogenous astaxanthin	36.1 g/L	Li et al. (2020)
Sugar cane and RSM method	*B. thuringiensis*	Shake flask fermentation	Sugar cane yeast extract	Study the effect of different carbon and nitrogen resources, media optimization by RSM method	2.38 g/L	Shukla and Mishra (2013)
Cane molasses and HSC strategy	*S. albulus* PD-1	168 h fed-batch fermentation	Cane molasses waste cells	Pretreated cane molasses and hydrolysate of *Strepyomyces* cells (HSC)	PDAP: 5.2 g/L	Xia et al. (2014b)
					ε-PL: 20.6 g/L	
Remnant glucose from levan production	*S. albulus* IFO 14147	72 h fed-batch fermentation	Sugar yeast extract	Remaining sugar solution fermentation levan as carbon source	4.37 g/L	Shih et al. (2011)
Economical medium and ANN method	*S. albulus* M-Z18	192 h fed-batch fermentation	Glycerol, fish meal corn steep liquor	Optimization low-cost carbon and nitrogen resource by artificial neural network (ANN)	35.24 g/L	Ren et al. (2015a)
Repeated-batch SSF strategy	*S. albulus* PD-1	192 h fed-batch fermentation	Wheat bran glucose	Solid-state fermentation (SSF)	75.51 mg/gds	Xu et al. (2017)
		Repeat eight times		Wheat bran (2: 1, w/w)		
				Glucose (4%, w/w)		
				Initial moisture content (65%)		

a Represent the carbon/organic nitrogen resource in medium.

## Downstream Processes

Downstream processing has been reported to occupy an important place in production, and its cost can reach 50–70% of the total production cost in some industrial biotechnology fields (Wei H. et al., 2018). Most ε-PL purification routes are based on ion-exchange strategies. The first ε-PL purification route consisted of cation ion-exchange adsorption, activated-carbon decoloration, organic-solvent precipitation, and gel chromatography (Shima and Sakai, 1981). Zhu et al. (2016) deveploped the purification process by applications of strong cation (HZB-3B) and weak cation (D155) resins, leading to high desorption efficiency and overall recovery of 97.57 and 94.49%, respectively. However, most ε-PL purification processes are for applications requiring chemical structure identification, and organic solvents used in the above methods could hide some dangers for future industrial production. In 2010, Jia et al. (2010) used an ultra-filtration technique with 2 and 5 kDa cut-offs to fractionate ε-PL, the largest advantage of this approach was that the ε-PL product could be controlled to ensure a relatively high molecular weight (3.2–4.5 kDa) product. However, the high cost of freeze drying remains a problem for industrial applications.

Combinatorial approaches for higher ε-PL purity and recovery have been developed in consideration of the strengths and weaknesses of the above methods. For example, Bankar et al. (2014) loaded culture broth sequentially onto a chromatographic column (Amberlite IRC 50), followed by ultrafiltration (20 and 5 kDa), solvent precipitation, and gel permeation chromatography, finally obtaining products with a purity of 97.58% and ε-PL yield of 90.42% under the optimal conditions. For large scale purification, Chen et al. (2018) sequentially used 30 kDa ultra-filtration, ion-exchange adsorption, macroporous resin and 1 kDa ultra-filtration for protein removal, purification, decolorization and desalting. To further improve the efficiency of ε-PL purification, cation ion-exchange resin (IRC-50) with NH_4_^+^ has been suggested to offer the highest adsorption capability and desorption ratio for ε-PL extraction, and ε-PL purity was enhanced from 90.2 to 97.10% under optimal conditions (Chen J. et al., 2019). Furthermore, another method combing ion-exchange resin (Amberlite IRC-50), macroporous adsorption resin (SX-8), and gel column chromatography (Sephadex G-25) can also extract ε-PL from the culture broth successfully (Chen X. et al., 2019).

Precipitation is a rapid and relatively simple method for high recovery of ε-PL. Katano et al. (2012) first used the tetraphenylborate anion to precipitate and separate ε-PL from a 100-ml fermentation broth, rapidly obtaining ε-PL at a 95% recovery rate. These workers subsequently developed the dipicrylamine anion (a yellow anionic dye) for precipitating polycationic ε-PL (Katano et al., 2015). Metal-chelate affinity precipitation was also used to separate and purify ε-PL, which afforded electrophoretic purity with recovery as high as 98.42% (Li et al., 2017a). Although precipitation processes can obtain high ε-PL purification from small volumes of fermentation broth containing ε-PL, the heavy use of acetone can introduce food-safety problems in industrial production.

Recently, aqueous two-phase extraction was developed as an environmentally friendly method for selective separation of ε-PL from fermentation broth. To this end, Sequeira et al. (2021) constructed four kinds of ionic liquid aqueous biphasic systems by using different ionic liquids with polypropylene glycol, consequently reaching 93.42% extraction efficiency for ε-PL in culture broth. An aqueous biphasic system can also be combined with ultra-filtration for large scale ε-PL purification. In this system, ε-PL from culture broth was subjected to triplicate extraction by ethanol/ammonium aqueous two-phase system, followed by desalting by ultra-filtration, which together provided new avenues for ε-PL purification (Chen et al., 2020). Although it resulted in a high purity (92.39%) ε-PL product at an acceptable recovery ratio (87.72%), this extraction method may cause some the environmental and economic problems.

Overall, many operations such as filtration, extraction, drying and sub-packaging are required for downstream ε-PL processing, which can cumulatively increase the cost. To date, the drawbacks in downstream processing have posed one of the major obstacles limiting the commercialization of ε-PL.

## Recent Advances in Applications of ε-PL

At present, ε-PL is mainly used as an effective food preservative to control pathogenic microorganisms and food spoilage due to its safety and strong antimicrobial activity. Many recent applications of ε-PL in biomedicine industry, such as dressing material and suture, also depend on its antimicrobial capacity (Fursatz et al., 2018; Peng et al., 2020). As a consequence, the mechanisms underlying its antimicrobial effects has become a hot research topic in recent years (Shu et al., 2021). To elucidate its antimicrobial mechanism, we focus on describing the action mode of ε-PL on various microorganism, including its effect on the cell wall, cell membrane, cytoplasm, gene and protein expression as well as the metabolic pathways. On this basis, recent applications of ε-PL in food and pharmaceutical industries were briefly summarized.

### Antimicrobial Property and Mechanisms of ε-PL

As a natural, cationic antimicrobial peptides, ε-PL is well known for its strong inhibition of bacteria, fungi, and viruses (Shima et al., 1984; Mookherjee et al., 2020). The strength of the inhibitory effect on cell growth by ε-PL is evaluated in Table 3. It is observed that in comparison with α-PL (6.4 kDa), ε-PL (3.2–3.8 kDa) was more effective against bacteria. Furthermore, both Gram-positive and Gram-negative bacteria have been found to be sensitive to naturally occurring ε-PL (1–8 μg/ml), but a similar inhibitory effect is only observed in yeast and filamentous fungi at much higher concentrations (>128 μg/ml).

**TABLE 3:** Antimicrobial activity of ε-PL.

	MIC (μg/ml)^a^	
	ε-PL	α- PL
**Bacteria**		
*Escherichia coli*	1–2	4–8
*Bacillus subtilis*	1	4
*Micrococcus tetragenus*	32	
*Pseudomonas aeruginosa*	3	16
*Staphylococcus aureus*	4	16
*Pseudomonas putida*	2	8
*Proteus vulgaris*	2	32
*Serratia marcescens*	8	>64
*Aerobacter aerogenes*	8	32
*Alcaligenes faecalis*	8	>64
*Bacillus brevis*	3	8
*Bacillus cereus*	16	32
*Arthrobacter simplex*	8	
*Arthrobacter globiformis*	8	16
*Corynebacterium xerosis*	2	4
*Micrococcus aurantiacus*	8	8
*Micrococcus roseus*	3	
*Micrococcus lysodeikticus*	2	
*Micrococcus luteus*	4	4
*Mycobacterium tuberculosis*	32	
**Yeasts**		
*Saccharomyces cerevisiae*	128	
*Saccharomycopsis lipolytica*	256	
*Candida utiliz*	128	
*Candida tropicalis*	128	
**Molds**		
*Aspergillus niger*	>256	
*Penicillium chrysogenum*	256	
*Penicillium urticae*	>256	
*Fusaruim oxysporum*	>256	
*Candida albicans*	128	

a This table is modified from the reports from Shima et al. (1984).

**FIGURE 6:**
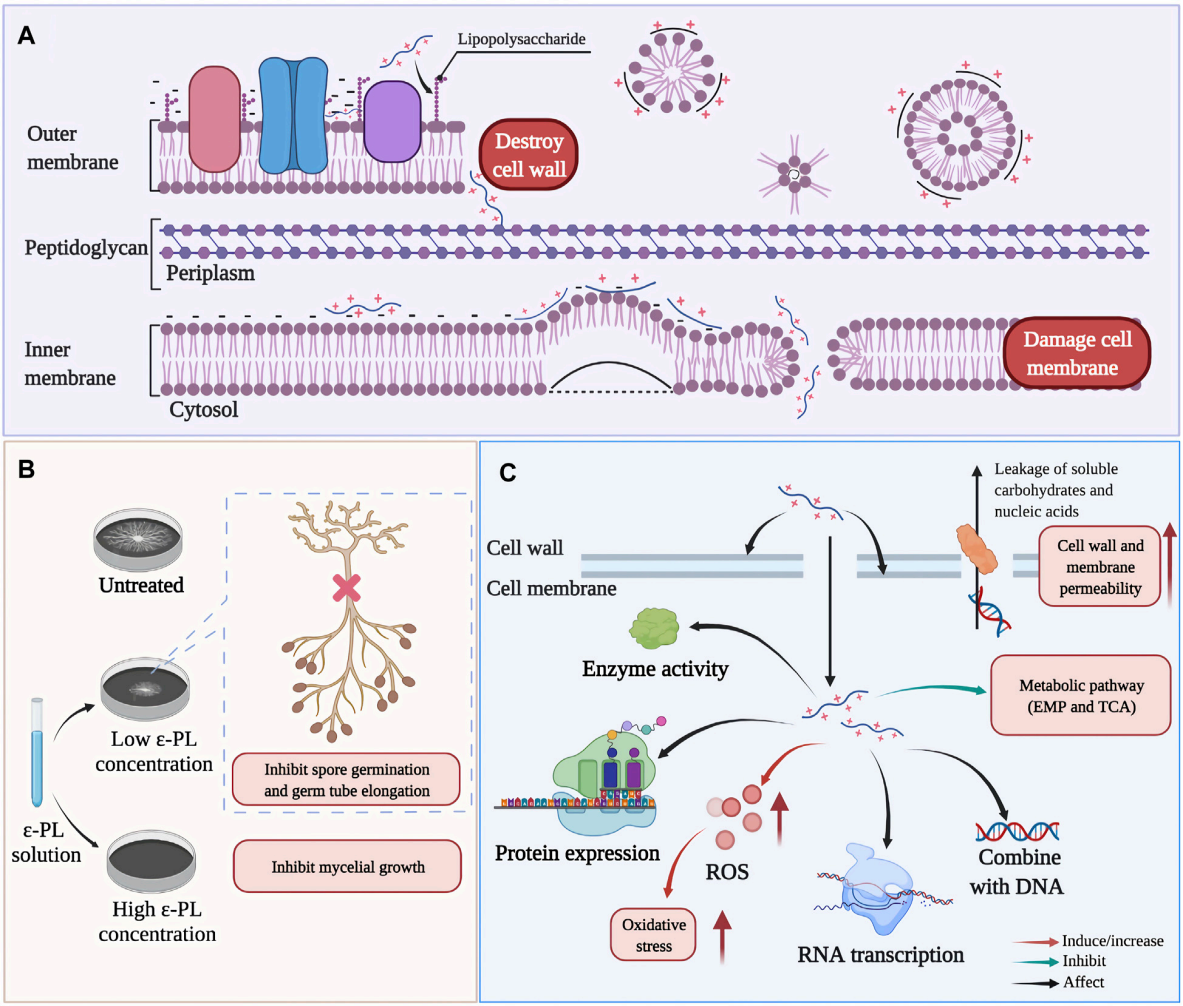
Possible antimicrobial mechanisms of ε-PL. **(A)** the action of ε-PL on cell structure of *Escherichia coli* O157:H7 (Ye et al., 2013). **(B)** The inhibition of ε-PL on spore germination and germ tube elongation (Liu Z. et al., 2020). **(C)** Summary of main antibacterial actions of ε-PL on different microorganism, such as *E.coli, Shewanella putrefaciens, Staphylococcus aureus, Saccharomyces cerevisiae, Salmonella Typhimurium* (Ye et al., 2013; Tan et al., 2019; Hou et al., 2020; Shen et al., 2020; Hou et al., 2021).

Multiple reports have described that ε-PL inhibits the growth of various microorganisms by affecting the integrity and permeability of their cell walls and cell membranes (Hyldgaard et al., 2014; Wei M. et al., 2018; Lan et al., 2019; Su et al., 2019; Liu H. et al., 2020; Dou et al., 2021). For example, in *Escherichia coli* O157:H7 (Figure 6A), the interactions between ε-PL and phospholipid groups result in distorting and shriveling of the cell membrane in a non-specific, carpet-like mechanism, in which the membrane is forced to fold in on itself, contributing to vesicle/micelle and toroidal pore formation (Hyldgaard et al., 2014). Additionally, in *Alternaria alternata* and *Botrytis cinerea*, ε-PL was also observed to inhibit spore germination and germ tube elongation (Figure 6B), leading to abnormal morphological development (Jiao et al., 2020; Li et al., 2019; Liu H. et al., 2020).

Deeper research has revealed that upon entry into the cytoplasm, ε-PL stimulates the production of ROS, and can affect cellular responses to oxidative stress and self-defense, ultimately retarding respiration, impeding cell viability, and thus potentially inducing cell death. For example, treatment with ε-PL can induce the accumulation of intracellular ROS and elevate expression of oxidative stress genes (*sodA* and *oxyR*) in *E. coli* O157:H7, while decreasing transcription of virulence genes (*eaeA* and *espA*) and SOS response genes (*lexA*) (Ye et al., 2013). In addition to killing some pathogens of humans, animals, or plants, ε-PL can also trigger host defense signaling in the infected organisms. For example, in jujube fruit infected with grey mold (caused by *B. cinerea*), the expression of defense response (respiratory burst oxidase homolog, RBOH) gene was induced by ε-PL treatment, while in tomato (*Solanum lycopersicum*), ε-PL treatment led to activation of specific antifungal defense-associated transcripts (β -1,3- glucanase, chitinase, and other PR proteins) (Sun et al., 2017; Li et al., 2019). Recently, high-throughput omics-based techniques greatly advanced our understanding of the impacts of ε-PL on cellular metabolism and related physiological effects. Proteomic screens have revealed that exposure to ε-PL can down-regulate the expression of protein-synthesis components in *Shewanella putrefaciens* (Hou et al., 2021). Similarly, genome-wide DNA microarray analysis showed that ε-PL could inhibit the ability of *Salmonella typhimurium* to colonize food by down-regulating the expression of genes necessary for biofilm formation (Shen et al., 2020). In addition, metabolomics analysis also demonstrated that ε-PL-induced stress resulted in the inhibition of primary metabolic pathways (mainly glycolysis and the tricarboxylic acid cycle (TCA cycle)) in *Saccharomyces cerevisiae* and *Staphylococcus. aureus* (Bo et al., 2014; Tan et al., 2019) (Figure 6C).

### Recent Applications of ε-PL

Given the natural and highly effective cationic antimicrobial properties, ε-PL and its derivatives have various applications in food, biomedicine and biomaterial industries. Although naturally occurring ε-PL has a bitter flavor, ε-PL is widely used as food preservative to improve the quality and shelf life of numerous foods, including starch-based foods, seafood, dairy products, as well as fruits and vegetables (Tuohetisayipu et al., 2019). For instance, Li et al. (2017b) found that alginate-based edible coating containing 0.05% ε-PL significantly inhibited the growth of yeast and mould, while maintained the green color, total chlorophylls content, and antioxidant capacity of the fresh-cut kiwifruit. In addition, the combination of ε-PL with other food additives can further improve the antimicrobial effect (Sun et al., 2018). For example, minimal inhibitory concentration of ε-PL and nisin (another natural food preservative) towards spoilage *Lactobacillus plantarum* were and 75 and 0.468 mg/L, respectively, while the combined use of ε-PL and nisin at much smaller concentrations (9.375 and 0.05 mg/L) can completely inhibit the growth of spoilage *L. plantarum* (Bortolotto et al., 2021). In another research, the combined use of ε-PL (200 mg/L) and chitooligosaccharide (400 mg/L) also showed much stronger antifungal activity against *B. cinerea* than the use of ε-PL (200 mg/L) or chitooligosaccharide (400 mg/L) alone (Sun et al., 2018).

Furthermore, ε-PL has also been adopted as an antimicrobial emulsifier by conjugation with dextran through the Maillard reaction. The conjugate obtained from this process has characteristics superior to those of many commercial emulsifiers, such as Sunsoft SE-11 and Q-18S (Li et al., 2014). As a dietary agent, ε-PL can reportedly prevent high-fat diet-associated weight gain by inhibiting lipase activity, which results in suppression of dietary fat absorption from the small intestine (Tsujita et al., 2006; Tsujita and Takaku, 2009). The lipid-lowering effects and structural stability of ε-PL can be further improved by formation of a complex between cationic ε-PL and anionic pectin through electrostatic interactions (Song et al., 2017). Very recent research has shown that ε-PL could affect nutrient utilization (i.e., proteins, lipids, and fiber) by regulation of the gut microbiota in Ningxiang pigs (Zhang et al., 2020).

In the biomedical industry, ε-PL was originally used as an effective antitumor and antiviral adjuvant for introducing high levels of serum interferon in monkeys, chimpanzees, and humans (Levy et al., 1969; Wakamoto et al., 2007). However, recent studies have shown that ε-PL can serve as an effective drug carrier due to its high biocompatibility in target cells, high drug loading capacity, wide range of molecular sizes appropriate for specific needs, as well as non-toxicity and excellent biodegradability in target cells (Chen et al., 2021). For example, Wu et al. (2020) used metformin hydrochloride (MetHCl) as a model drug, and designed dissolving tablets consisting of a carboxymethylcellulose/poly-L-lysine/tripolyphosphate complex (CMC/PLL/TPP). This formulation showed a high drug loading efficiency (85.76%) and rapid drug dissolution and release profile (i.e., complete release within 10 min in simulated saliva) in the environment. These results implied that this ε-PL complex could be potentially used as a viable drug delivery system (Wu et al., 2020). In addition, ε-PL and its complexes, such as pluronic grafted dendritic α, ε-PLs (DPL-PF127) and ε-PL-sodium alginate nanoparticles, have been used as novel carriers for delivery of non-viral genes and vaccines (Dung et al., 2016; Yuan et al., 2018). Another recent report described a magnetic mesoporous silica/ε-PL nanomotor-based complex with strong potential for application in treatment of heavy metal poisoning due to its high capacity for removal of excessive lead ions from blood (Liu J. N. et al., 2020).

ε-PL has also been used in the production of other biomaterials, such as antimicrobial hydrogels for wound healing dressings, biosensor for direct detection of triglycerides in whole blood and as a fiber-based biosorbents for microbial inactivation and dyes removal (Wang R. et al., 2017; Xu T. et al., 2018; Jiang and Zhou, 2021). Furthermore, ε-PL has been reported to function as an ideal protectant for enzymes because its linear structure provides sufficient space for modification of enzyme surfaces by its amino groups, thus enhancing accessibility to the active site (Huang et al., 2019).

## Conclusions and Future Prospects

ε-PL is an industrially attractive molecule due to its great potential as an antibacterial food preservative, therapeutic gene or drug carrier, enzyme stabilizer, and component of cosmetics. Since its discovery in 1977 by Shima and Sakai, research on ε-PL has spanned 44 yr (Shima and Sakai, 1977). Current research has enhanced the biosynthetic capabilities of ε-PL-producing strains through cell engineering approaches including mutagenesis, genome shuffling, ribosome engineering, and genetic engineering. Many studies have also attempted to enhance the efficiency of ε-PL biosynthesis and reduce costs through development of optimized industrial production strategies and utilization of agri-industrial wastes. Of course, many problems such as regulation of the complex metabolic networks, low productivity and conversion rate for ε-PL biosynthesis, have hindered its broad commercial adoption.

The development of different strategies is thus necessary to achieve stable, high-yielding strains and to enhance the biosynthesis of ε-PL. First, genetic and metabolic strategies can be used to enhance the biosynthetic pathway activity while inhibiting or eliminating competing pathways. Multi-omics comparative analyses between high-yield (obtained by conventional methods) and low-yield strains can also provide abundant candidate sites for targeted mutagenesis and directed strain evolution by metabolic engineering. In addition, with the rapid development of synthetic biology, the minimal genome required for ε-PL production could be determined and assembled, thus possibly enabling ε-PL biosynthesis in different or non-model microorganisms, facilitated by advanced tools for genetic modification. Industrial microorganisms are subject to a variety of environmental stress-inducing factors and adverse conditions, and adaptive evolution may be an effective strategy to improve their tolerance to these environmental stresses. ε-PL biosynthesis could also be potentially improved through directed evolution of ε-PL synthetase, enhancing its acid tolerance and catalytic efficiency under low pH. In addition, since high concentration of ATP is required for industrial ε-PL synthesis, it may be fruitful to regulate ε-PL biosynthesis through enhanced oxygen or ATP availability, through genetic engineering, or novel strategies for oxygen delivery, and new bioreactors. In view of its sophisticated ATP synthesis and metabolic capabilities, novel ε-PL-producing strains can be engineered with a *Streptomyces* chassis to produce other high-ATP-requirement products, such as hyaluronic acid (Yoshimura et al., 2015).

Overall, as a high-value product, ε-PL has a wide range of prospective industrial applications that could benefit from its safe and bioactive properties. With further understanding of its biomanufacturing process, more advanced biotechnologies will be used to enhance the biosynthetic capacity for this product, reducing the cost of biomanufacturing and accelerating its industrialization, ultimately promoting the future commercial application of ε-PL.

## Data Availability

The datasets generated for this study are available on request to the corresponding author.
